# Medical Societies

**Published:** 1853-10

**Authors:** 


### EDITORIAL.

*Medical Societies.*

The organization of District and County Medical Societies furnishes the most satisfactory evidence of the advancement of our science; while at the same time it enables the members of the profession to act together in opposing quackery and in disseminating truth in reference to the great questions of life and death.

The physician, perhaps more than any other man, needs independence in thought and action; the individuality of man is extended to his labors; his habits of investigation, and his application of therapeutical rules are in most instances essentially his own; each, therefore, in the discharge of his daily round of duties is accumulating a fund of experience, valuable, it is true, to himself, but equally or more valuable to others, from the fact, that his observations were made, so to speak, from a different stand point, his facts have been examined in a different light. It results from this, that in our individual observations, there are necessarily errors, but these errors are corrected by the observations of others: the truth is the mean.

We are gratifyed to see in our own North-West the spirit of progress manifesting itself in organization, by a desire to become acquainted with each others views, and to avail ourselves of each others experience.

During the last few months, as will be seen by referring to our Journal, Medical Societies have been organised in Illinois, Wisconsin, Iowa, and even Minnesota.

There are scattered throughout this vast extent of territory men of sterling worth, of cultivated minds and extension of knowledge, but the demands on their time, and the physical labor which they have to undergo in a country practice, leaves them little leisure or strength for recording their observations, and especially is this the case when they have no particular inducement to do so. In their local societies these men have a plan to work; they are stimulated by meeting each other, and are armed with each others strength.

It will be remembered that the next meeting of the American Medical Association is to be held in St. Louis. Our Eastern brethren look to that meeting as an exponent of the talent of the profession in the West. Some of them think that the interests of the Association will suffer by going beyond the bounds of civilization almost, but if the members of the profession in the West do their duty, the meeting in St. Louis will show, that, though west of the mountains, we are not beyond the reach of light. We speak honestly when we say, we believe that no section of the United States is supplied with better practical men—men who, in addition to a knowledge of the theories of disease and of the methods of cure, are possessed of better common sense in the application of therapeutical rules, than in our North West. Let then the profession continue to organize, and when organizations are once formed, let them be kept up.

Many of the papers read in the local societies are valuable and should be preserved; two of the County Societies of Pennsylvania have recently united in publishing a journal made up of their transactions. In cases where this is not deemed advisable, communications of importance may be preserved by sending them to the publishers of medical journals already in existence. If all County and District Societies would adopt this plan, their transactions might be preserved in a form convenient for consultation by every member, while at the same time the professional public would be equally benefitted by the results of their experience and observation. J.

